# Exploring Green Creativity: The Effects of Green Transformational Leadership, Green Innovation Climate, and Green Autonomy

**DOI:** 10.3389/fpsyg.2022.686373

**Published:** 2022-03-14

**Authors:** Qamaruddin Maitlo, Xiuting Wang, Yan Jingdong, Ishfaque Ahmed Lashari, Naveed Ahmad Faraz, Nazim Hussain Hajaro

**Affiliations:** ^1^Department of Business Administration, Sukkur IBA University, Sukkur, Pakistan; ^2^School of Management, Wuhan University of Technology, Wuhan, China

**Keywords:** green creativity, leadership, climate, green innovation climate, green autonomy

## Abstract

None of the studies published in the extant literature has discussed the role of green innovation climate and green autonomy concerning green creativity and this study aims to offer these two novel constructs. By introducing the componential theory of creativity, this study explores green transformational leadership (GTL), green innovation climate, and green autonomy as antecedents of green creativity. The authors employed structural equation modeling (SEM) to analyze survey-based data collected from automotive firms in China. Data were collected from employee-supervisor working in the automotive industry located in Liaoning province, China. The findings reveal that GTL directly and indirectly via green innovation climate partially mediates the green creativity of employees in China. Moreover, green autonomy moderates the relationship between green innovation climate and green creativity. This pivotal contribution suggests that automotive business enterprises should develop GTL to nurture a green innovation climate and offer green autonomy for the green creativity of employees. The above antecedents of green creativity may enable business firms to gain a competitive advantage by innovating green products and practices.

## Introduction

The natural resource-based view postulates that business firms would be dependent upon nature in the future and only those business firms would survive, which would have environmental sustainability, as their core business strategy and rooted in capabilities (Hart, 1995). Recent studies have reported that the global community, through the Paris accord, has agreed to work on policies and strategies in phasing out business firms that burn fossil fuels and pollute the environment (Qureshi et al., 2016). Thus, such organizations must be transformed or replaced with green firms and the automotive industry is not an exception. Due to higher carbon and nitrogen oxide emissions, the automotive industry has taken initiatives to become green (UCSUSA, 2019). For instance, Tesla, Chevrolet, BMW, Nissan, Ford, Volkswagen, and many other firms are racing to innovate green and clean vehicles (Enelx, 2019). In order to innovate green and clean, companies have to incorporate green and clean philosophy in their core strategies (Awan et al., 2019). Greening the internal mechanisms also provides a competitive advantage of green and energy-efficient products and services heterogeneity (Chen and Chang, 2013; Mittal and Dhar, 2016; Awan et al., 2019). Besides, green and clean production enhances resource efficiency and decreases environmental pollution (Hart, 1995). Green creativity (GCT) is vital for sustainable green and clean production (Awan et al., 2019; Li et al., 2020). GCT refers to “the development of new ideas about green products, green services, green processes, or green practices that are judged to be original, novel, and useful” (Chen and Chang, 2013). GCT is crucial for developing unique green ideas that may lead to green innovation and production (Mittal and Dhar, 2016). In the automotive industry, green creative behavior is the foremost step in the green innovation process, as it is crucial for environment-friendly innovation (Jia et al., 2018). Although various factors influence green creative behavior, such as green passion (Jia et al., 2018), green organizational identity (Mittal and Dhar, 2016), green intrinsic, and extrinsic motivation (Li et al., 2020), the role of green leadership is yet underresearched, particularly in the automotive industry.

A few recent studies explicated that green creative performance is highly dependent on leaders who have a green vision (Chen and Chang, 2013; Li et al., 2020). For instance, Juliet Davenport, CEO of Good Energy, has the vision to help Britain become 100% renewable by supplying 100% electricity devices and equipment (Townsend, 2018). Leaders, such as Elon Musk (CEO of Tesla), Sarah, a voice of green energy (Founder Director of Ashden), and many others, have shared their vision and inspiration for climate change (Townsend, 2018). However, leadership in Asia is seen behind in the race of climate protection and cleaner production. Scholars maintain that green transformational leadership (GTL) with characteristics develops inspiration and motivation to innovate green products and services (Amabile and Pratt, 2016; Li et al., 2020). Although a few studies have investigated the relationship between GTL and green creative outcomes and innovation (Chen and Chang, 2013; Li et al., 2020), it remains silent in the Chinese automotive industry.

The theory of creativity posits that individuals’ creativity can be influenced by the social or work climate (Martins and Terblanche, 2003; Amabile and Pratt, 2016). It is suggested that organizational climate can stimulate the creative behavior of employees even when subordinates are basically motivated and possess relevant skills (Amabile and Pratt, 2016; Li et al., 2020). This study aims to conceptualize the green innovation climate (GIC), which provides flexibility and freedom to think in green creative ways to unleash green innovation. Building on the creativity theory, it is argued that the GIC helps to direct and channel activities toward green innovation (Scott and Bruce, 1994; Amabile and Pratt, 2016). For instance, Elon Musk, the CEO of Tesla, removes the hierarchy barrier in the organization to listen to any green idea that can help to innovate green vehicles and solve environmental problems (Tesla, 2019). GIC guarantees that employees’ green performance is recognized and rewarded for green creative ideas. Moreover, a GIC ensures that subordinates are equipped with the resources and necessities required for GCT (Sarros et al., 2008; Jaiswal and Dhar, 2015). Nevertheless, it is observed that prior studies have only focused on the green psychological climate, green self-efficacy, green dynamic capabilities (Chen et al., 2014; Norton et al., 2017; Joshi and Dhar, 2020), and in the GCT literature (Faraz et al., 2021). This study, in hand, intents to advance the literature on GCT by exploring the mediation role of GIC between green transformational and GCT.

A quantitative meta-analysis revealed that the climate for innovation and autonomy is the best predictors for employees’ creativity (Hunter et al., 2007). Employees need autonomy to imagine green creative ideas for green and clean products and practices. For instance, Tesla provides high-task autonomy to think green creative ideas and share directly with leadership via email or any mode of communication (Fournier, 2019; Tesla, 2019). Thus, autonomy to generate green novel ideas and share with leadership without any barrier motivates employees to think differently. The theory of self-determination supports the argument that autonomy elevates motivation connected with creativity in any context (Deci, 2015). When employee are given freedom they fell proud and engage in creative pro environmental tasks (Liu et al., 2011; Deci, 2015; Li et al., 2020) and (Ying et al., 2020). Preceding studies have investigated the general autonomy construct in organizational setting (Zhou, 1998; Hunter et al., 2007; Deci, 2015). However, green autonomy (GA), which would be more effective in predicting GCT in the sustainable production context, is absent in existing literature. Therefore, this study identifies GA as a moderating construct that ensures freedom and choice to think and share any untried green ideas for sustainable production in the automotive industry.

**FIGURE 1:**
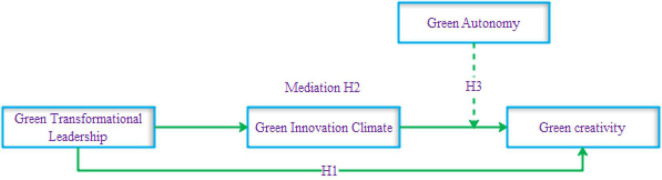
Conceptual framework.

This study intends to offer two novel constructs: GIC and GA. Then, only a few studies have explored the impact of GTL on green creative behavior in Indian (Mittal and Dhar, 2016) and Taiwanese (Chen et al., 2014) industries using the resource-based view model and ability-motivation-opportunity theory. This study explores this relationship through the theoretical lens of the componential theory of creativity in the Chinese automotive industry, which is yet to be investigated. Another novel contribution of this study to GCT is to examine the effect of GIC between GTL and creativity in the cleaner production setting. Last, the authors also aim to evaluate the role of GA (moderator) impacting the relationship between GIC and green creative behavior in the environmental context, as shown in Figure 1.

## Theoretical Foundation and Hypotheses Development

### Green Transformational Leadership and Green Creativity

Green transformational leadership refers to a leadership style that involves motivating and inspiring employees to achieve environmental objectives and exceed the expected green creative performance level (Chen and Chang, 2013). A few recent studies have established that GTL plays a significant role in stimulating green creative outcomes (Chen and Chang, 2013; Mittal and Dhar, 2016; Jia et al., 2018). GTL has four dimensions: charisma, individualized consideration, inspirational motivation, and intellectual stimulation (Bass, 1985). Charisma enables leaders to show the green vision to their team members and set high standards for green innovation (Chen and Chang, 2013). Leadership through inspirational motivation imbues a sense of team spirit and motivation to achieve green innovation (Li et al., 2018). Individualized consideration helps leaders to guide employees by understanding and recognizing their needs and motivations to foster GCT (Zhou et al., 2018). Last, through intellectual stimulation, leaders raise the cognitive ability of personnel to think from different paradigms and perspectives for the creative solutions of old problems (Avolio et al., 1999).

Green creativity is vital for sustainable green and clean production (Awan et al., 2019; Li et al., 2020). GCT is crucial for developing unique green ideas that may lead to green innovation and production (Mittal and Dhar, 2016). Although various factors influence green creative behavior, such as green passion (Jia et al., 2018), green organizational identity (Mittal and Dhar, 2016), and green intrinsic and extrinsic motivations (Li et al., 2020), the role of green leadership is yet underresearched, particularly in the automotive industry. Innovation development cannot take place without new ideas and new ideas cannot emerge without creativity (Wyer and Xu, 2010). Creativity is process in which new ideas are generated that lead to producing new approaches that result in taking newer actions. This process of converting novel ideas into productive and useful business practices are the foundation of innovation to take place in any organization (Wyer and Xu, 2010). Creativity is a necessary step within the innovation process because creativity is a starting point for innovation (Amabile et al., 1996). Referring to Amabile (1998), the GCT is described as “the development of new ideas about green products, green services, green processes, or green practices that are judged to be original, novel, and useful.”

Therefore, organizational creativity or organization GCT is the primary impetus for organizational innovation or innovation produced and reflected in products and services of an organization (Halbesleben et al., 2003). There is sufficient evidence available in prior literature that the success of new products is based on team creativity that facilitates the whole process of product development (Chang and Hsu, 2010). Product performance and further augmentation are also direct outcome of creativity (Hunt and Morgan, 1995). It is argued by Cooper (1979) and Deshpandé et al. (1993) that unique ability of creative teams can respond to consumer needs effectively and can also respond to ever-changing market dynamics in a way to capitalize the opportunities posed by otherwise uncertain situation. Ford in 1996 claimed that assessment of creativity is a degree to which creative ideas are useful and original to end users.

For firms, team creativity or group creativity plays a significant role to enhance innovation process and create innovative culture (Yoon et al., 2010). Hence, prior research indicates that the creativity and in context “green” philosophy, the GCT influences green product development, green team performance, green culture in organization, and green behavior of employees working in green culture (Cooper, 1979; Smith and Reinertsen, 1992; Amabile et al., 1996; Griffin, 1997).

Employees under GTL engage in GCT, which leads to green innovation. In order to achieve GCT, leadership should actively listen to employees, share knowledge, and provide a framework of growth and opportunities through long-term vision (Mittal and Dhar, 2016; Jia et al., 2018). Recent studies have established the connection between GTL and GCT (Chen and Chang, 2013; Mittal and Dhar, 2016; Jia et al., 2018). For instance, Mittal and Dhar (2016) found that GTL raised the green creative behavior of employees for conserving the environment in tourist hotels. Another two studies revealed a positive relationship between GTL and **GCT** of employees in the technology industry in order to conserve the environment from contamination (Chen and Chang, 2013; Li et al., 2020). Having this in mind, the authors propose the following hypothesis.

H1: Green transformational leadership is positively related to GCT in the Chinese automotive industry.

### Mediation of Green Innovation Climate

Organizational climate represents the firm’s characteristics such as attitudes, behaviors, and feelings that exist independently irrespective of the perceptions and understandings of the members of the organization (Ekvall, 1996). In other words, an innovative climate exists when organizations have a certain degree of trust, say, and openness among the members, risk-taking mentality, and commitment to perform creative tasks (Ekvall, 1996). For instance, employees receive signals concerning innovative behavior in an innovative climate. By understanding and using these signals, employees often respond to expectations and regulate their innovative behavior to raise their self-satisfaction and pride (Scott and Bruce, 1994).

The proponents of the innovative climate included two work-climate dimensions: (1) support for innovation and (2) resource supply (Scott and Bruce, 1994; Jaiswal and Dhar, 2015). Support for innovation refers to support for team members or employees to perform independently, searching for creative and innovative ideas, and respect for diversity and creative work in an organization (Ren and Zhang, 2015). For instance, in an experimental study (Amabile and Pratt, 2016), it revealed that subjects who received the rewards as a bonus had higher creativity than those of the other groups, which implies that rewarding and recognizing creative performance nurture a climate for innovation. Therefore, support and encouragement among team members can yield green creative performance. Provision of resource supply includes equipment, facilities, and the time considered critical to creativity and innovation (Amabile and Pratt, 2016). For instance, Amabile posited that access needed to appropriate resources such as materials, information, finance, and facilities has a moderate impact on employee’s creativity, which is another manifestation of the green innovative climate (Amabile and Pratt, 2016).

Extending the study of Scott and Bruce (1994), the authors advance a new concept of GIC, which refers to “a set of employees perceptions about the work environment that encourages risk-taking behavior for green products and practices, allocates adequate resources, and provides challenging work climate for GCT and innovation at an organizational level.” Investigating the relationships between GTL and GCT is not new to the scholars (Chen and Chang, 2013; Li et al., 2020). For instance, studies contended that GTL helps to enhance employees’ GCT in different industries and under different conditions through its characteristics (Chen and Chang, 2013; Mittal and Dhar, 2016; Li et al., 2020). Moreover, researchers also maintained that leaders develop a conducive climate, whereby they encourage employees by providing adequate support and resources for creativity (Scott and Bruce, 1994; Jaiswal and Dhar, 2015). For instance, Amabile and Pratt (2016) argue that leaders should motivate personnel to develop novel ideas, recognize their creative work, and provide support and means to reach the goals for innovation across the firms.

Therefore, the authors maintain that a GIC, wherein employees find support, encouragement, and adequate resources for green products, services, and processes from leadership, would stimulate GCT. A study held on the hotel industry in India found that innovation climate mediates the relationship between transformational leadership (TFL) and here it stand for Creativity (CRT) (Jaiswal and Dhar, 2015). Similarly, a study wherein 32 Taiwanese companies participated maintained that innovation climate positively and partially mediates the relationship between TFL and CRT (Jung et al., 2003). Based on the above discussion, it is postulated that GTL nurtures an innovation climate, which increases GCT. Thus, the authors formulate the hypothesis.

H2: Green innovation climate mediates the relationship between GTL and GCT.

### Moderating Role of Green Autonomy

Green autonomy refers to “the freedom given to employees in performing their green and proenvironmental tasks” (Ekvall, 1996). Referring to self-determination theory, “the need for autonomy represents individuals’ inherent desire to feel volitional and to experience a sense of choice and psychological freedom when carrying out an activity” (Van den Broeck et al., 2010, p. 982). Some scholars believe that autonomy can enable employees to think creatively and raise innovative ideas (Deci, 2015). They further argue that an autonomy-supported environment tends to raise the intrinsic motivation of employees; thereby, they curiously think about problems, generate ideas, and test for implementation (Krause, 2004). On the other hand, micromanagement or jobs with little discretion hinder employee’s ability to be creative. A meta-analysis concluded that the concentration of decision-making authority and less dispersion of power tend to diminish innovation in an organization (Damanpour, 2012). Therefore, providing autonomy and freedom to employees can help them to undertake different experiments and procedures for better green creative performance.

Componential theory of creativity maintains that organizational climate and autonomy can have a significant effect on employees’ motivation to think creatively and study quotes Einstein’s statement, “It is a very grave mistake to think that the enjoyment of seeing and searching can be promoted by means of coercion and a sense of duty.” Autonomy is a crucial determinant of creativity and employees tend to accomplish creative tasks when they perceive more freedom (Amabile and Pratt, 2016). Furthermore, scholars revealed a positive relationship between innovation climate and environmental performance such as GCT (Jung et al., 2003; Jaiswal and Dhar, 2015). In this study, it is posited that GA moderates the relationship between GIC and GCT. In the meta-analysis (Hughes et al., 2018), it revealed that autonomy and discretion provided by leadership tend to stimulate creativity in firms.warwarwa.

Building on the theoretical support and arguments, it is advanced that in an automotive industry where GIC promotes GCT, GA, and freedom to think green ideas and solutions to environmental problems may foster green creative ideas to produce green and clean products and services. In light of the foregoing, the authors hypothesize:

H3: Employees are more likely to come up with green creative ideas in a GIC when they have higher GA.

## Materials and Methods

### Context and Participants

The authors collected data from the automotive industry located in Liaoning province, China. The choice of the industry, province, and country has several reasons: First, the automotive industry in Liaoning province contributes 12.7% share of gross industrial product and it is under immense pressure to innovate clean and green products and services to curtail environmental hazards (HKTDC, 2019). Second, this industry is going through sharp incremental and radical innovation as China plans to accelerate the electrification of the four-wheelers (Rathi, 2019). Last, air pollution in northern China is sharply rising and 70% of this pollution is contributed by industries that failed to comply with environmental regulations and standards (Wong, 2017). Therefore, China has initiated a war on pollution, cracking down on firms violating environmental laws.

### Measures

The survey, which was employee-based and self-administered, was first developed in the English language; then, it was translated into the Chinese language using the back-translation technique. The authors also availed the services of two bilingual experts to ensure conversion and content quality (Brislin, 1970). The scales ranged from 1 to 5, where 1 represented strongly disagree and 5 represented strongly agree.

#### Green Transformational Leadership

A six-item survey of GTL developed by Chen and Chang (2013) was used. The reliability and validity of the measures were also tested by various researchers (Chen and Chang, 2013; Mittal and Dhar, 2016). The sample items are: “(1) our leader inspires us with the environmental plans and (2) our leader provides us a clear environmental vision to follow.” All the items reflect more than 0.655 correlation values with each other thus, showing the reflective nature of the construct.

#### Green Creativity

The authors adopted a six-item scale developed by Chen and Chang (2013) for measuring the green creative behavior of employees at work and the sample items are: “(1) He/she suggests new ways to achieve environmental goals and (2) He/she proposes new green ideas to improve environmental performance.” The scale’s reliability is 0.950, satisfying the criteria of reliability (Hair et al., 2014). Moreover, the items of GCT indicate reflective nature, as they have a higher than 0.685 correlation with each other.

#### Green Innovation Climate

This study offers an original idea, “GIC,” for which the authors refer to the innovation climate proposed by Scott and Bruce (1994) and further validated in a recent study by Jaiswal and Dhar (2015). The eight-measurement items of the construct were modified considering the environmental context (Scott and Bruce, 1994). The items are: “(1) GCT is encouraged here; (2) Around here, people are allowed to try to solve the old problems in different greenways; (3) This organization can be described as flexible and continually adapting to green change; (4) This organization is open and responsive to environmental change; (5) There is adequate time available to pursue green creative ideas here; (6) This organization gives free time to pursue green creative ideas during the workday; (7) The reward system here encourages green innovation; and (8) This organization publicly recognizes those whose ideas are green and innovative.” Last, all the items of the construct indicate a reflective nature, as they have higher correlation values (higher than 0.507).

#### Green Autonomy

This study offers a novel notion by extending the scope of autonomy to GA for which the authors refer to Broeck (Van den Broeck et al., 2010) and define GA as “individuals’ inherent desire to feel volitional and to experience a sense of choice and psychological freedom when carrying out a proenvironmental activity.” In addition, the authors refer to other studies (Van den Broeck et al., 2010; Deci and Ryan, 2015) to measure GA that include five items, which were altered considering the environmental context: “(1) I feel like I can be myself at my job doing in greenway; (2) The tasks I have to do at work are in line with the environment and what I really want to do; (3) I feel free to do tasks in the greenway; (4) I feel free to do things at work in greenway; and (5) In my job, I feel forced to do green tasks I do not want to do.” All the measures are reflective because they show higher than 0.605 correlation values with each other.

### Procedures

For data collection, the authors contacted twenty automotive firms located in Liaoning province during July to December, 2019. At the initial stage, the authors gave a presentation to top management and briefed them about the significance and the academic purpose of this study. Moreover, they were assured of sharing the findings and practical implications of this study. Later on, after receiving consent, employees from R&D and production departments were informed about the survey, which was self-administered. Two research assistants instructed employees about the purpose of the survey and helped to understand different survey sections. They also elaborated about green constructs employed in this study and what they mean. Employees were assured that the survey is highly confidential and anonymous and they do not have to provide their names or other identities. They were requested to complete section (A) of the survey and return it to the authors. Survey (A) included questions related to GTL, GIC, and GA. Supervisors were provided section (B) of the survey about GCT of their employees working in the same department to corroborate and match data. A total of 600 questionnaires with numbers coded were handed over to employees and supervisors and out of which only 307 (51.2%) questionnaires were found valid for analysis. In management sciences, especially with dyadic design, a response rate above 50% is generally considered as acceptable (Baruch and Holtom, 2008). In total, 105 supervisors participated and responded about the GCT of their 307 subordinates who responded about GTL, GIC, and GA. Various studies suggest that when a model has four arrows leading to endogenous construct, that study should have a minimum of 40 samples (Hair et al., 2014; Ali Memon et al., 2017); however, this study has more than 300 sample size and it implies that this study satisfies the sampling criteria.

We performed factor analysis to ensure items that indicate required reliability and validity. Table 1 shows that each variable can be classified into only one factor and has an accumulated percentage of variance explained (Li et al., 2020). Additionally, for checking common method bias (CMB), the authors used Harman’s one-factor test approach (Harman, 1976). The six items of GTL, eight items of GIC, five items of GCT, and five items of GA were entered in SPSS using the principal component factor analysis technique. The first factor in the model explained 31.1% of the variance (Harman, 1976). Thus, the authors confirmed that there is no CMB issue in the analysis. Socially desirable responding (SDR) refers to the problem when respondents compromise the validity of the survey (Nederhof, 1985). To deal with this issue, the surveyors promised secrecy regarding data, so that respondents fill in the survey with honesty (Nederhof, 1985). Moreover, researchers did not include any question, which could have disclosed the identity of employees. Thus, the research article bears no issues of common method variance (CMV) and socially desirable responding (SDR).

**TABLE 1:** Constructs’ reliability, factor loading, average variance extracted (AVE), and accumulated explained variance.

Constructs/Items	Factor loading	VIF values	Cronbach’s alpha	Composite reliability	AVE	Number of factors	Accumulation percentage (%) of explained variance
Green autonomy (GA)			0.917	0.932	0.734	1	75.1
GA1	0.898**	3.063					
GA2	0.857**	2.920					
GA3	0.890**	2.200					
GA4	0.828**	3.204					
GA5	0.806**	2.983					
Green creativity (GC)			0.950	0.960	0.799	1	79.9
GC1	0.880**	4.216					
GC2	0.901**	4.595					
GC3	0.849**	3.194					
GC4	0.915**	5.212					
GC5	0.923**	5.048					
green transformational leadership (GTL)			0.943	0.953	0.773	1	77.8
GTL1	0.901**	3.467					
GTL2	0.878**	3.450					
GTL3	0.895**	3.788					
GTL4	0.881**	5.209					
GTL5	0.855**	4.470					
GTL6	0.865**	4.375					
Green innovation climate (GIC)			0.939	0.948	0.695	1	70.0
GIC1	0.844**	3.373					
GIC2	0.862**	3.628					
GIC3	0.851**	2.977					
GIC4	0.821**	2.859					
GIC5	0.912**	5.738					
GIC6	0.814**	2.843					
GIC7	0.794**	4.182					
GIC8	0.764**	3.604					

N = 307. **p < 0.01.

## Data Analysis and Results

The authors leveraged the structural equation modeling (SEM) technique using SmartPLS version 3.2.8 software to test the hypotheses. Partial least squares (PLS-SEM) is widely used for the analysis of prediction oriented the studies such as when questionnaires are modified into a new context (Henseler et al., 2014); besides, this technique is preferred when a study offers new constructs or modifies old constructs and for prediction purposes (Hair et al., 2014). Moreover, this software is extensively used in marketing, human resource management, psychology, and other disciplines, as it can handle non-normal data, complex models where mediation and moderation are involved, and other problematic issues (Hair et al., 2014). The authors followed a two-step approach. First, the authors establish reliability and validity. Subsequently, the authors predict structural relationships among latent independent and dependent constructs with mediation and moderation.

Table 2 indicates the mean, SD, correlation of variables, and other demographic details. Although education, experience (in years), and age of employees (in years) are control variables in this study, as they impact individuals’ creativity (Amabile and Pratt, 2016), these variables are insignificantly correlated with GCT, GTL, GIC, and GA. Moreover, the supervisor’s education, gender, age, and company tenure (in years) are insignificantly correlated with employees’ creativity. The average age of employees is above 30 years and the supervisors’ age is approximately 50 years. Most of the employees and supervisors working in the automotive industry are male and employees have a mean experience above 3 years. Moreover, GTL, GIC, and GA are positively and significantly correlated with GCT.

**TABLE 2:** Means, SD, and correlation values.

	*N* = 307	Mean	*SD*	1	2	3	4	5	6	7	8	9	10	11	12
1	Education	2.39	0.540	1											
2	Experience	3.18	2.314	0.027	1										
3	Age	30.24	5.650	−0.124*	0.135*	1									
4	Supervisor’s age	50.03	4.427	0.098	–0.069	0.175**	1								
5	Supervisor’s gender	1.70	0.457	–0.041	0.094	0.053	0.011	1							
6	Supervisor’s education	2.39	0.660	0.098	0.151**	0.193**	0.008	–0.005	1						
7	Company tenure	14.38	4.958	0.000	0.059	0.044	–0.033	−0.119*	0.110	1					
8	Gender	1.60	0.491	–0.038	0.150**	0.079	–0.028	0.745**	–0.005	0.000	1				
9	GTL	2.982	1.223	–0.021	0.014	0.095	0.009	–0.045	0.008	0.055	0.038	1			
10	GA	3.415	1.087	0.098	0.032	–0.057	0.017	–0.059	0.022	–0.057	–0.052	–0.033	1		
11	GIC	3.462	1.006	0.084	0.051	–0.059	0.025	–0.064	0.006	–0.065	–0.059	0.254**	0.238**	1	
12	GCT	3.425	1.222	0.016	0.020	–0.096	–0.009	–0.013	–0.022	0.003	0.066	0.320**	0.218**	0.315**	1

**p < 0.01, *p < 0.05; GTL, Green transformational leadership; GA, Green autonomy; GIC, Green innovation climate; GCT, Green creativity.

*Gender: 1 = female (40.4%), 2 = male (59.6%).*

*Supervisor’s gender: 1 = female (29.6%), 2 = male (70.4%).*

*Education: 1 = Diploma (0%), 2 = undergraduate (63.2%), 3 = Post graduate (34.2%), 4 = Ph.D. (2.6%).*

*Supervisor’s Education: 1 = Diploma (5.5%), 2 = Undergraduate (54.1%), Post graduate (36.2%), 4 = Ph.D. (4.2%).*

### Measurement Analysis

Table 1 indicates that Cronbach’s alpha and composite reliability values are above the required values (0.7), reflecting higher consistency and reliability (Hair et al., 2014). Factor loading values are higher than 0.764 and average variance extracted (AVE) values are also higher than 0.5 criteria; thus, establishing constructs’ convergent validity (Hair et al., 2014). Moreover, discriminant validity establishes that the constructs are empirically unique and different from each other (Henseler et al., 2014). The authors also verified discriminant validity using the heterotrait-monotrait ratio (HTMT) approach, in which case the HTMT values should be lower than 0.85 thresholds (Henseler et al., 2014). In this study, the HTMT values of constructs are below 0.338, exhibiting higher discriminant validity. Thus, data in this analysis meet the criteria of reliability and validity for conducting the structural analysis.

### Structural Analysis

A structural model is used to analyze the relationship between endogenous and exogenous latent constructs. The authors first confirmed that there is no multicollinearity issue in the model by checking variance inflation factor (VIF) values of items that are lower than 5, excluding four items that have below 5.7 (see Table 3). *R*^2^ indicates predictive accuracy (Hair et al., 2014) and this study indicates lower-to-moderate predictive accuracy, as an *R*^2^-value range from 2.5 to 3 in the final model # 3. The *R*^2^-value of model 3 indicates a 28.2% variance explained by the exogenous constructs. *F*^2^-values of the models such as 0.02, 0.15, and 0.35 reflect small, moderate, and large size effects, respectively (Hair et al., 2014). *F*^2^-values in our analysis for the final model represent small-to-medium effects.

**TABLE 3:** Structural model analysis and hypotheses testing.

	Relationship	Path value	*P*-value	*T*-value	*R*^2^-value	*F*^2^-value	Hypotheses
Model # 1 without mediation	GTL→ GCT	0.358	0.000	8.678	0.128	0.147	
Model # 2 (with mediation of GIC)	GTL→ GCT (Total effect)	0.350	0.000	7.773	0.190	0.085	
	GTL→ GCT (Direct effect)	0.273	0.000	5.312			
	GTL→ GCT (Indirect effect)	0.076	0.001	3.475			
	GTL→ GIC	0.282	0.000	4.881	0.079	0.086	
	GIC→ GCT	0.271	0.000	5.540		0.083	
Model # 3 with moderation of GA	GTL→ GCT (H1) Total effect	0.339	0.000	7.654	0.282	0.084	Supported
	GTL→ GCT (H4) (Indirect/Mediation effect)	0.078	0.001	3.341			Supported
	GTL→ GCT (Direct effect)	0.261	0.000	5.076			
	GIC→ GCT (H3)	0.276	0.000	4.910		0.081	Supported
	GTL→ GIC (H2)	0.282	0.000	4.979	0.079	0.086	Supported
	Moderation/Interaction effect of GA-GIC on GCT (H5)	0.196	0.009	2.612		0.055	Supported
	GIC→ GCT	0.215	0.000	5.024		0.059	

*GTL, Green transformational leadership; GCT, Green creativity; GIC, Green innovation climate; GA, Green autonomy.*

#### Goodness of Fit

A good model fit reflects that the model is plausible and parsimonious. Although a myriad of studies provides model fit in the research, PLS-SEM does not recommend it. PLS-SEM technique provides *R*^2^ and *F*^2^-values in place of model fit to evaluate the exploratory power of the model (Hair et al., 2014; Li et al., 2020). However, for satisfaction, the authors calculated the model fit using Tenenhaus et al. (2005) approach. They have recommended the following equation [GoF = √(AVE × *R*^2^)] to calculate the GoF, which other researchers also have used. The cutoff values for the GoF are GoF small = 0.1; GoF medium = 0.25; and GoF large = 0.36 (Farooq et al., 2018). This study yields a 0.474 value for the GoF and indicates a very good model fit.

To check *T*-values and significance of the path values, the authors ran bootstrapping with a 5,000 resample (Hair et al., 2014). All the relationships, including direct effects, indirect effects, and moderator, were significant at *p*-value 0.01, as shown in Table 3. This research indicates that GTL has a substantial impact on the green creative behavior of employees in all the models and predictive accuracy *R*^2^-value for GCT increases when mediator (GIC) and moderator (GA) involve.

#### Mediation and Moderation

The authors, first, confirmed the significance of the direct relationship between exogenous construct GTL and endogenous latent construct GCT of employees working in the automative industry. Later, the mediator was inserted into the model and the indirect effect of GIC was found to be significant at a *p*-value of 0.001. Last, the authors’ measured variance accounted for (VAF) 21.7% showing partial mediation (Hair et al., 2014). In the 3rd model, the authors inserted moderator, GA, and created an interaction effect with the GIC using the product indicator approach (Hair et al., 2014). The moderation of GA was also found significant at a *p*-value of 0.009. Therefore, this empirical study establishes that GIC mediates the relationship between GTL and GCT and GA moderates the relationship between GIC and GCT.

## Discussion and Implications

The increasing significance of environmental concerns and green innovation has gained the attention of research scholars to search out the mechanisms that foster GCT and innovation. This study can be considered one of the pioneer studies examining the role of GIC as a mediator and GA as a moderator.

Consistent with preceding studies (Mittal and Dhar, 2016; Li et al., 2020), this study confirms that green transformational leaders influence the green creative behavior of subordinates in the automotive industry of China. Thus, leaders with green vision and inspiration are more effective in innovating green products and services, as observed in European and Western business firms (Rimmer, 2018; Townsend, 2018; Tesla, 2019). Another possible explanation might be that employees in the Chinese automotive industry are more leadership oriented in a collective culture and follow them accordingly.

This empirical investigation examined the role of GIC as a mediator between GTL and GCT, which is absent in the past studies. This study first indicates, in congruence with the prior research (Jaiswal and Dhar, 2015), GIC directly affects the GCT of employees and, second, it stimulates the green creative behavior of employees in the automotive industry as a mediator between GTL and GCT. This study conveys that GTL can effectively engage their followers in green creative behavior by providing them enough support, resources, and appreciation.

This study further reveals that GA moderates the relationship between GIC and GCT, implying that employees with higher autonomy for GCT tend to have higher green creative behavior under an elevated GIC. On the other hand, from the analysis, the authors infer that if autonomy for GCT is low in the organization, even a high level of GIC may not elevate the green creative behavior of employees, thus ending with low GCT in companies. Our finding suggests that granting freedom and autonomy for sharing green creative ideas boosts employees’ morale, and as a result, GCT increases, which is revealed as a secret of GCT in Tesla company (Rimmer, 2018; Fournier, 2019). Therefore, the authors propose that the automotive industry in China should follow the same approach and provide autonomy to raise green creative ideas for green and sustainable production.

### Theoretical Contribution

This study adds to the literature on GTL, GIC, GA, and GCT of employees working in the automotive industry using creativity theory. This study has offered novel constructs: GIC and GA, which is the study’s main contribution. The second contribution of this study is that a GIC mediates between GTL and GCT in the automotive industry of China, which is absent in the extant literature (Jaiswal and Dhar, 2015; Chen and Hou, 2016).

**FIGURE 2:**
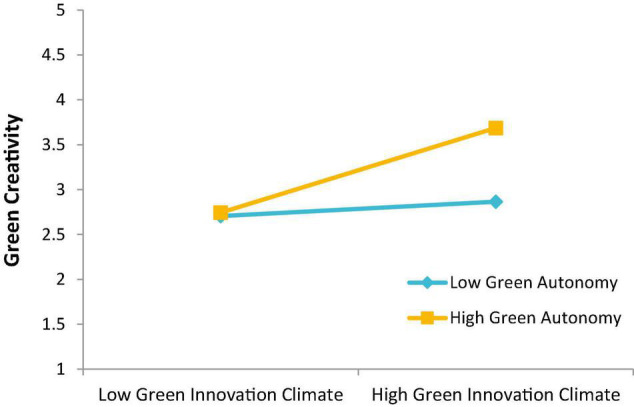
Two-way interaction graph.

The third contribution of this study is that it offers a novel construct of GA, which strengthens the relationship between GIC and GCT of personnel when freedom is higher for green creative behavior of employees under the circumstances when GIC is also higher (see Figure 2). Thus, GIC and GA are important constructs that play an interactive role in raising the green creative behavior of employees in the automotive industry. Thus, this empirical investigation contributes to creativity literature in a green environment where leadership fosters employees’ GCT for green and sustainable innovation. Altogether, this study suggests that the automotive industry can curtail environmental hazards and save the environment by using the mechanism of GCT, GIC, and GTL. Therefore, the authors propose that organizations develop green leadership and GIC to make green and clean production through GCT possible.

### Managerial Implications

Following are the practical implications for managers in this study.

First, our findings suggest that enhancing GTL can increase GCT and also raise GIC. If companies would like to enhance their GIC, they should incorporate the concepts of green innovation, GTL, and GCT into their long-term corporate strategies.

Second, employees of orgnizations must be educated and made aware of the green practices. They must be given autonomy and space to express their creative ideas and, hence, improve green production. This, in long run, will also produce quality leadership sensitive to GIC, GTL, and GCT. Third, companies need to enhance GCT across all the layers of organization, so that new ideas may emerge and help companies to innovate.

Fourth, since green climate or green culture has become an effective approach to develop differentiation and positioning strategies nowadays, firms should exploit green innovative practices to differentiate and to position their products in order to seize new green markets. Our findings provide some practical implications for management, employees, and firms working in the automotive industry. In order to foster GCT, firms should embed idiosyncratic characteristics of GTL to their leadership and management, which would elevate the green creative behavior of employees. Thus, following the data and literature, this study suggests that leaders in green organizations should provide green vision and inspiration to employees and then remove the communication barrier to nurture the flow of green creative ideas across the organization. Green transformational leaders who have a mindset of green innovation and creativity uplift their subordinates through the development of GIC and supply resources and other requirements needed for GCT in organizations considering ecological and environmental strategies. Hence, the automotive industry can reduce and curtail environmental hazards by adopting GIC and GTL because leadership through innovation supportive climate can develop employees’ green creative behavior leading to environment-friendly innovation.

Among many other challenges, GTL has to ensure that employees are given a high degree of autonomy to generate and share green ideas for green innovation. In this regard, management should ensure employees’ freedom to think, freedom to decide, and freedom to choose alternative approaches that could lead to green innovation. Thus, green creative ideas should be allowed to flow from bottom to top management, even the company’s CEO. Tesla Company considers GA and freedom to share green ideas as one of the culture’s core values (Fournier, 2019). Thereafter, employees provided with a GIC and GA would endeavor to generate green ideas to develop eco-friendly products and services.

Last, automotive firms in China, especially in Liaoning provinces, should understand their responsibility toward the environment; they should also propagate environmental concerns and issues to their management and employees to achieve a high level of green innovation. This study, through its findings, provides a mechanism through which firms and employees can take benefits to attain a high level of green innovation.

### Limitations and Future Research

This study was conducted in Liaoning province of China. The authors confronted with some of limitations while conducting this study.

First, it was suggested by many firm workers and supervisors during initial briefings for the study that the most relevant group who possess knowledge and understanding about green environment and GCT would be R&D and production department. Therefore, the focus of data collection was restricted to mainly these departments.

Second, a couple of automotive firms refused to provide data regarding their leadership attributes and innovation climate, even after using references and approaches. Third, this study can be extended to other industries such as the furniture industry, construction industry, and the food industry because these industries are progressively expanding and causing pollution in the northern region of China. Another limitation of this study is that it is a cross-sectional study (one time). Therefore, researchers may involve longitudinal and multilevel approaches to understand GIC, GTL, and GCT in the fast-growing industries of China and other countries. Last, future studies may consider employing GIC and GA variables for further validation and analysis purposes in a different context for achieving eco-friendly innovation. Also, the two dimensions of mediating variable can be tested separately to see the individual affect of resources supply and management support.

## Conclusion

The central point of this study is that manufacturing industries, specifically the automotive industry, can take advantage of GCT and innovation when they link their management philosophy to the green management philosophy and embody green strategies to the company’s strategies.

This empirical research presents two novel notions, such as GIC and GA that influence the green creative behavior of employees. Moreover, GTL directly promotes GCT and indirectly through partial mediation of GIC. This study also revealed that GA strengthens the impact of GIC on the green creative performance of employees. By taking advantage of this study, the automotive firms should design training programs related to green strategies and corporate social responsibility to cultivate a GIC and transformational leadership to boost employees’ behavior for green and clean production.

## Data Availability Statement

The raw data supporting the conclusions of this article will be made available by the authors, without undue reservation.

## Author Contributions

QM conceptualized the main idea and research framework of this research manuscript, contributed to expertise in the related field brought rich insights into framing this study and designing the methodology and research questions for the said project. All authors contributed to the article and approved the submitted version.

## Conflict of Interest

The authors declare that the research was conducted in the absence of any commercial or financial relationships that could be construed as a potential conflict of interest.

## Publisher’s Note

All claims expressed in this article are solely those of the authors and do not necessarily represent those of their affiliated organizations, or those of the publisher, the editors and the reviewers. Any product that may be evaluated in this article, or claim that may be made by its manufacturer, is not guaranteed or endorsed by the publisher.
